# [2-(1,3-Dithio­lan-2-yl­idene)-5-(4-methyl­phen­yl)-3-oxopent-4-enoato-κ*O*]tri­phenyl­tin(IV)

**DOI:** 10.1107/S1600536808015754

**Published:** 2008-06-07

**Authors:** Ze-Min Mei, Wei Fang

**Affiliations:** a Department of Chemistry, BaiCheng Normal College, BaiCheng 137000, People’s Republic of China

## Abstract

In the title compound, [Sn(C_6_H_5_)_3_(C_17_H_17_O_3_S_2_)], the Sn^IV^ atom adopts a distorted tetra­hedral SnC_3_O geometry. A short intra­molecular Sn⋯O contact of 2.793 (2) Å also occurs.

## Related literature

For related literature, see: James *et al.* (1992 ▶).
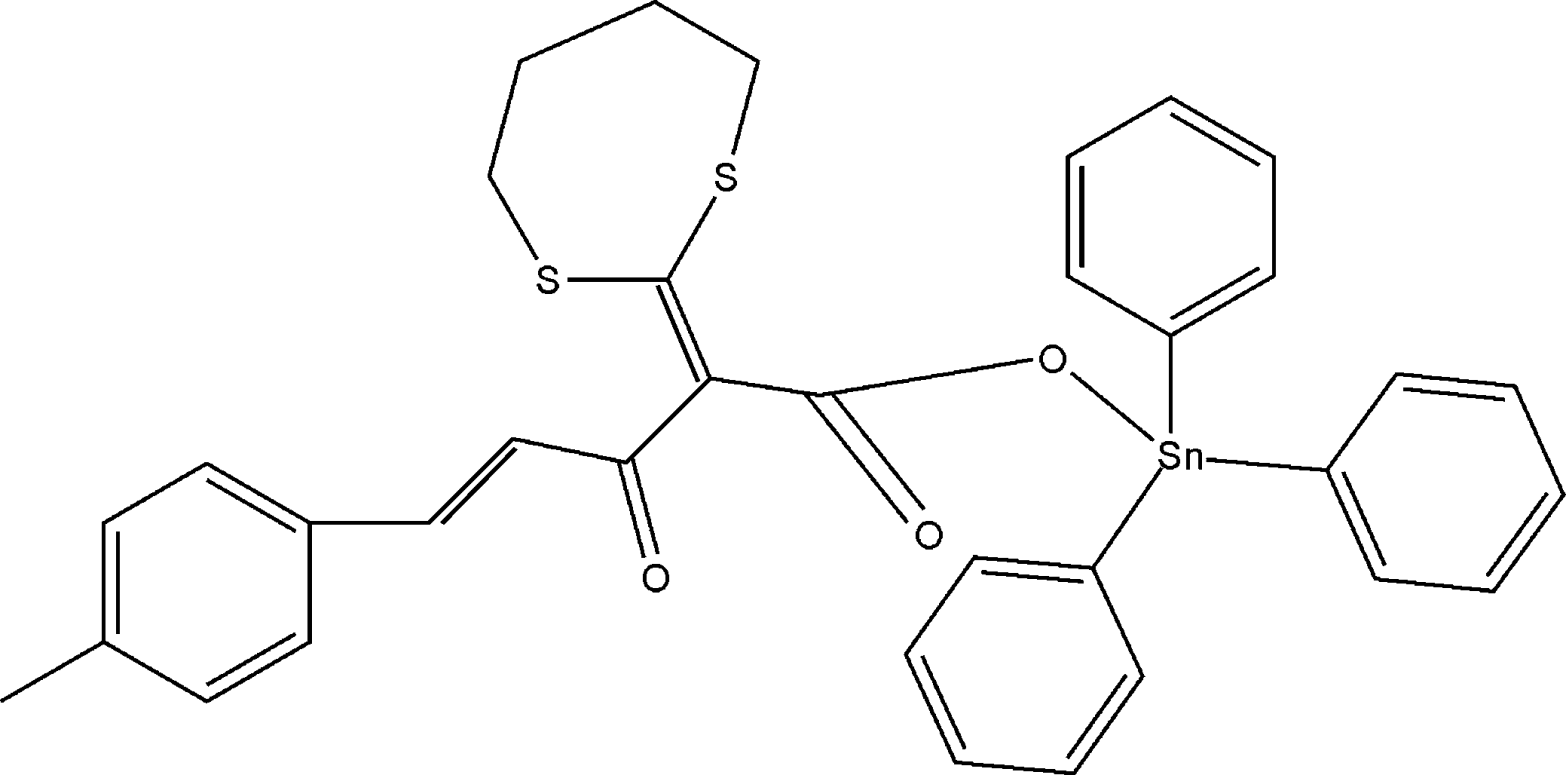


## Experimental

### 

#### Crystal data

[Sn(C_6_H_5_)_3_(C_17_H_17_O_3_S_2_)]
*M* *_r_* = 683.42
Monoclinic, 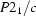

*a* = 12.736 (3) Å
*b* = 14.945 (3) Å
*c* = 17.733 (4) Å
β = 106.85 (3)°
*V* = 3230.4 (11) Å^3^
*Z* = 4
Mo *K*α radiation
μ = 0.95 mm^−1^
*T* = 292 (2) K
0.43 × 0.21 × 0.08 mm

#### Data collection

Bruker APEX diffractometer
Absorption correction: multi-scan (*SADABS*; Bruker, 2002 ▶) *T*_min_ = 0.793, *T*_max_ = 0.943
7230 measured reflections
5654 independent reflections
4186 reflections with *I* > 2σ(*I*)
*R*_int_ = 0.017

#### Refinement

*R*[*F* ^2^ > 2σ(*F* ^2^)] = 0.029
*wR*(*F* ^2^) = 0.066
*S* = 0.91
5654 reflections
370 parameters
H-atom parameters constrained
Δρ_max_ = 0.32 e Å^−3^
Δρ_min_ = −0.25 e Å^−3^

### 

Data collection: *SMART* (Bruker, 2002 ▶); cell refinement: *SAINT* (Bruker, 2002 ▶); data reduction: *SAINT*; program(s) used to solve structure: *SHELXS97* (Sheldrick, 2008 ▶); program(s) used to refine structure: *SHELXL97* (Sheldrick, 2008 ▶); molecular graphics: *SHELXTL* (Sheldrick, 2008 ▶); software used to prepare material for publication: *SHELXTL*.

## Supplementary Material

Crystal structure: contains datablocks global, I. DOI: 10.1107/S1600536808015754/hb2734sup1.cif

Structure factors: contains datablocks I. DOI: 10.1107/S1600536808015754/hb2734Isup2.hkl

Additional supplementary materials:  crystallographic information; 3D view; checkCIF report

## Figures and Tables

**Table 1:** Selected bond lengths (Å)

Sn—O1	2.0716 (19)
Sn—C13	2.121 (3)
Sn—C1	2.128 (3)
Sn—C7	2.135 (3)

**Fig. 1.**
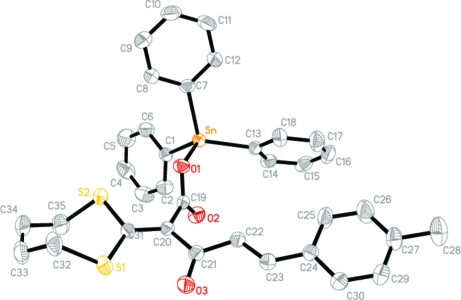
The molecular structure of (I) with displacement ellipsoids drawn at the 50% probability level (H atoms omitted for clarity).

## supplementary crystallographic information

### Comment

Organotin compounds have been extensively studied owing their biological activities and varied structures (e.g. James *et al.*,  1992). Here, we present the synthesis and structure of the title molecular complex, (I), (Fig. 1).

The tin atom in (I) is coordinated to three carbon atoms from three phenyl groups and one oxygen atom from a carboxylate group (Table 1). A short Sn···O contact of 2.793 (2)Å also occurs, so the coordination of the carboxylate group could also be described as very asymmetric bidentate although the C-O bond lengths are very different [1.224 (3) and 1.300 (3)Å], suggestive of charge localisation.

### Experimental

A mixture of Ph_3_SnOH (1.0 mmol) and 2-[1,3]dithiolan-2-ylidene-3-oxo-5-(4-methelphenyl)-pent-4-enolic acid 2 (1.0 mmol) in toluene (20 ml) was refluxed for 3 h and water released in the reaction was removed azeotropically by a Dean-Stark apparatus. The reaction mixture was then cooled and toluene removed by a rotary evaporator. The resulting solid product was recrystallized with ethanol to give colourless slabs of (I).

### Refinement

All H atoms were placed geometrically (C—H = 0.93-0.97 Å) and refined as riding with *U*_iso_(H) fixed at 0.08Å^2^.

### Crystal data

**Table:**

[Sn(C_6_H_5_)_3_(C_17_H_17_O_3_S_2_)]	*Z* = 4
*M**_r_* = 683.42	*F*_000_ = 1392
Monoclinic, *P*2_1_/*c*	*D*_x_ = 1.405 Mg m^−^^3^
Hall symbol: -P 2ybc	Mo *K*α radiation λ = 0.71073 Å
*a* = 12.736 (3) Å	θ = 2.9–22.2º
*b* = 14.945 (3) Å	µ = 0.95 mm^−^^1^
*c* = 17.733 (4) Å	*T* = 292 (2) K
β = 106.85 (3)º	Slab, colourless
*V* = 3230.4 (11) Å^3^	0.43 × 0.21 × 0.08 mm

### Data collection

**Table:**

Bruker APEXII diffractometer	5654 independent reflections
Radiation source: fine-focus sealed tube	4186 reflections with *I* > 2σ(*I*)
Monochromator: graphite	*R*_int_ = 0.017
Detector resolution: 0 pixels mm^-1^	θ_max_ = 25.0º
*T* = 292(2) K	θ_min_ = 1.7º
ω scans	*h* = −1→15
Absorption correction: multi-scan(SADABS; Bruker, 2002)	*k* = −1→17
*T*_min_ = 0.793, *T*_max_ = 0.943	*l* = −21→20
7230 measured reflections	

### Refinement

**Table:**

Refinement on *F*^2^	Secondary atom site location: difference Fourier map
Least-squares matrix: full	Hydrogen site location: inferred from neighbouring sites
*R*[*F*^2^ > 2σ(*F*^2^)] = 0.029	H-atom parameters constrained
*wR*(*F*^2^) = 0.066	*w* = 1/[σ^2^(*F*_o_^2^) + (0.0285*P*)^2^] where *P* = (*F*_o_^2^ + 2*F*_c_^2^)/3
*S* = 0.91	(Δ/σ)_max_ = 0.001
5654 reflections	Δρ_max_ = 0.32 e Å^−^^3^
370 parameters	Δρ_min_ = −0.25 e Å^−^^3^
Primary atom site location: structure-invariant direct methods	Extinction correction: none

### Special details

**Table:**

Geometry. All e.s.d.'s (except the e.s.d. in the dihedral angle between two l.s. planes) are estimated using the full covariance matrix. The cell e.s.d.'s are taken into account individually in the estimation of e.s.d.'s in distances, angles and torsion angles; correlations between e.s.d.'s in cell parameters are only used when they are defined by crystal symmetry. An approximate (isotropic) treatment of cell e.s.d.'s is used for estimating e.s.d.'s involving l.s. planes.
Refinement. Refinement of *F*^2^ against ALL reflections. The weighted *R*-factor *wR* and goodness of fit *S* are based on *F*^2^, conventional *R*-factors *R* are based on *F*, with *F* set to zero for negative *F*^2^. The threshold expression of *F*^2^ > σ(*F*^2^) is used only for calculating *R*-factors(gt) *etc*. and is not relevant to the choice of reflections for refinement. *R*-factors based on *F*^2^ are statistically about twice as large as those based on *F*, and *R*- factors based on ALL data will be even larger.

### Fractional atomic coordinates and isotropic or equivalent isotropic displacement parameters (Å^2^)

**Table:**

	*x*	*y*	*z*	*U*_iso_*/*U*_eq_	
Sn	0.369434 (16)	0.896438 (14)	0.055072 (11)	0.04525 (7)	
S1	0.04717 (8)	0.68154 (7)	0.23041 (5)	0.0680 (2)	
S2	0.16556 (7)	0.85632 (6)	0.21982 (5)	0.0676 (2)	
O1	0.31329 (16)	0.80230 (14)	0.11995 (11)	0.0529 (5)	
O2	0.16982 (18)	0.80468 (14)	0.01464 (12)	0.0605 (6)	
O3	0.0657 (2)	0.57848 (15)	0.10596 (15)	0.0754 (7)	
C1	0.2679 (2)	1.0122 (2)	0.03678 (16)	0.0480 (7)	
C2	0.1598 (3)	1.0121 (2)	−0.0094 (2)	0.0654 (9)	
H2	0.1293	0.9594	−0.0342	0.080*	
C3	0.0962 (3)	1.0882 (3)	−0.0197 (2)	0.0789 (11)	
H3	0.0234	1.0865	−0.0504	0.080*	
C4	0.1402 (4)	1.1657 (3)	0.0153 (3)	0.0890 (13)	
H4	0.0976	1.2173	0.0086	0.080*	
C5	0.2478 (4)	1.1680 (3)	0.0608 (3)	0.0944 (13)	
H5	0.2777	1.2212	0.0849	0.080*	
C6	0.3114 (3)	1.0923 (2)	0.0708 (2)	0.0677 (9)	
H6	0.3845	1.0949	0.1009	0.080*	
C7	0.5206 (2)	0.91750 (19)	0.14472 (18)	0.0491 (7)	
C8	0.5215 (3)	0.9442 (2)	0.22032 (18)	0.0599 (8)	
H8	0.4553	0.9551	0.2310	0.080*	
C9	0.6177 (3)	0.9546 (3)	0.2790 (2)	0.0757 (11)	
H9	0.6164	0.9723	0.3291	0.080*	
C10	0.7161 (3)	0.9392 (3)	0.2644 (2)	0.0787 (11)	
H10	0.7814	0.9470	0.3042	0.080*	
C11	0.7180 (3)	0.9124 (3)	0.1916 (2)	0.0786 (11)	
H11	0.7849	0.9013	0.1821	0.080*	
C12	0.6215 (2)	0.9013 (2)	0.13137 (19)	0.0602 (8)	
H12	0.6240	0.8831	0.0818	0.080*	
C13	0.3984 (2)	0.8288 (2)	−0.04210 (16)	0.0473 (7)	
C14	0.3547 (3)	0.8590 (2)	−0.11887 (18)	0.0596 (8)	
H14	0.3090	0.9088	−0.1289	0.080*	
C15	0.3792 (3)	0.8153 (3)	−0.18026 (19)	0.0758 (11)	
H15	0.3493	0.8357	−0.2316	0.080*	
C16	0.4463 (4)	0.7428 (3)	−0.1666 (3)	0.0853 (13)	
H16	0.4633	0.7148	−0.2084	0.080*	
C17	0.4890 (4)	0.7108 (3)	−0.0920 (3)	0.0829 (12)	
H17	0.5331	0.6600	−0.0831	0.080*	
C18	0.4665 (3)	0.7542 (2)	−0.0297 (2)	0.0651 (9)	
H18	0.4973	0.7332	0.0213	0.080*	
C19	0.2141 (2)	0.77828 (19)	0.08166 (17)	0.0452 (7)	
C20	0.1567 (2)	0.71697 (19)	0.12296 (15)	0.0436 (7)	
C21	0.1364 (2)	0.62483 (19)	0.09161 (17)	0.0494 (7)	
C22	0.2053 (3)	0.59116 (19)	0.04431 (17)	0.0503 (7)	
H22	0.2623	0.6267	0.0383	0.080*	
C23	0.1887 (3)	0.5114 (2)	0.00984 (17)	0.0522 (7)	
H23	0.1314	0.4781	0.0183	0.080*	
C24	0.2480 (2)	0.4690 (2)	−0.03935 (17)	0.0502 (7)	
C25	0.3373 (3)	0.5064 (2)	−0.0554 (2)	0.0894 (13)	
H25	0.3645	0.5608	−0.0323	0.080*	
C26	0.3877 (4)	0.4655 (3)	−0.1050 (3)	0.1033 (16)	
H26	0.4478	0.4935	−0.1145	0.080*	
C27	0.3535 (3)	0.3857 (2)	−0.1407 (2)	0.0694 (10)	
C28	0.4098 (4)	0.3420 (3)	−0.1948 (2)	0.1017 (15)	
H28A	0.4700	0.3787	−0.1985	0.080*	
H28B	0.3585	0.3352	−0.2462	0.080*	
H28C	0.4367	0.2843	−0.1744	0.080*	
C29	0.2650 (3)	0.3485 (3)	−0.1252 (2)	0.0863 (12)	
H29	0.2381	0.2943	−0.1488	0.080*	
C30	0.2133 (3)	0.3887 (2)	−0.0754 (2)	0.0753 (10)	
H30	0.1533	0.3605	−0.0660	0.080*	
C31	0.1233 (2)	0.7476 (2)	0.18458 (15)	0.0476 (7)	
C32	0.0722 (3)	0.7268 (3)	0.32917 (18)	0.0852 (12)	
H32A	0.0642	0.6786	0.3637	0.080*	
H32B	0.1478	0.7468	0.3471	0.080*	
C33	−0.0004 (3)	0.8036 (3)	0.3391 (2)	0.0883 (13)	
H33A	−0.0742	0.7930	0.3054	0.080*	
H33B	−0.0035	0.8038	0.3932	0.080*	
C34	0.0353 (4)	0.8927 (3)	0.3209 (2)	0.0983 (15)	
H34A	0.1061	0.9054	0.3582	0.080*	
H34B	−0.0162	0.9366	0.3294	0.080*	
C35	0.0451 (3)	0.9053 (3)	0.2383 (2)	0.0832 (12)	
H35A	0.0450	0.9690	0.2276	0.080*	
H35B	−0.0195	0.8798	0.2012	0.080*	

### Atomic displacement parameters (Å^2^)

**Table:**

	*U*^11^	*U*^22^	*U*^33^	*U*^12^	*U*^13^	*U*^23^
Sn	0.04499 (11)	0.04695 (12)	0.04510 (11)	−0.00348 (11)	0.01511 (8)	−0.00145 (10)
S1	0.0732 (6)	0.0831 (6)	0.0592 (5)	−0.0096 (5)	0.0375 (4)	−0.0034 (5)
S2	0.0619 (5)	0.0686 (6)	0.0770 (6)	−0.0067 (5)	0.0276 (5)	−0.0286 (5)
O1	0.0483 (12)	0.0601 (13)	0.0505 (11)	−0.0097 (11)	0.0145 (10)	0.0038 (10)
O2	0.0680 (14)	0.0639 (14)	0.0470 (12)	−0.0052 (12)	0.0125 (11)	0.0064 (11)
O3	0.0801 (17)	0.0673 (16)	0.0954 (18)	−0.0277 (13)	0.0517 (15)	−0.0229 (13)
C1	0.0501 (18)	0.0473 (18)	0.0500 (16)	−0.0004 (15)	0.0199 (15)	0.0040 (14)
C2	0.060 (2)	0.062 (2)	0.069 (2)	0.0024 (19)	0.0090 (18)	0.0082 (18)
C3	0.062 (2)	0.078 (3)	0.093 (3)	0.008 (2)	0.017 (2)	0.024 (2)
C4	0.078 (3)	0.067 (3)	0.129 (4)	0.024 (2)	0.042 (3)	0.016 (3)
C5	0.097 (3)	0.054 (2)	0.134 (4)	0.005 (2)	0.036 (3)	−0.015 (2)
C6	0.057 (2)	0.062 (2)	0.082 (2)	−0.0036 (19)	0.0177 (18)	−0.0059 (19)
C7	0.0453 (17)	0.0444 (18)	0.0567 (17)	−0.0069 (14)	0.0134 (14)	−0.0024 (14)
C8	0.0530 (19)	0.069 (2)	0.0597 (19)	−0.0056 (18)	0.0192 (16)	−0.0092 (17)
C9	0.070 (2)	0.093 (3)	0.059 (2)	−0.010 (2)	0.0114 (19)	−0.014 (2)
C10	0.054 (2)	0.090 (3)	0.080 (3)	−0.008 (2)	−0.0017 (19)	−0.008 (2)
C11	0.0416 (19)	0.093 (3)	0.101 (3)	0.003 (2)	0.0200 (19)	−0.005 (2)
C12	0.0523 (18)	0.066 (2)	0.068 (2)	−0.0056 (18)	0.0252 (16)	−0.0061 (18)
C13	0.0487 (17)	0.0497 (17)	0.0466 (16)	−0.0084 (15)	0.0190 (14)	−0.0039 (14)
C14	0.0555 (19)	0.067 (2)	0.0544 (18)	−0.0163 (18)	0.0127 (16)	−0.0024 (16)
C15	0.088 (3)	0.094 (3)	0.0456 (18)	−0.037 (3)	0.0198 (19)	−0.011 (2)
C16	0.110 (3)	0.080 (3)	0.088 (3)	−0.041 (3)	0.063 (3)	−0.038 (2)
C17	0.101 (3)	0.057 (2)	0.108 (3)	−0.004 (2)	0.058 (3)	−0.015 (2)
C18	0.075 (2)	0.062 (2)	0.065 (2)	0.0032 (19)	0.0303 (19)	−0.0027 (18)
C19	0.0487 (17)	0.0416 (16)	0.0477 (17)	−0.0021 (14)	0.0178 (14)	−0.0101 (14)
C20	0.0422 (16)	0.0498 (17)	0.0384 (14)	−0.0042 (14)	0.0112 (12)	−0.0032 (13)
C21	0.0512 (17)	0.0511 (19)	0.0469 (16)	−0.0062 (15)	0.0159 (14)	−0.0015 (13)
C22	0.0530 (18)	0.0492 (19)	0.0521 (16)	−0.0087 (15)	0.0206 (14)	−0.0047 (14)
C23	0.0559 (19)	0.0492 (18)	0.0525 (17)	−0.0004 (16)	0.0173 (15)	0.0018 (15)
C24	0.0541 (18)	0.0438 (17)	0.0527 (17)	0.0005 (15)	0.0155 (15)	0.0005 (14)
C25	0.116 (3)	0.059 (2)	0.123 (3)	−0.029 (2)	0.080 (3)	−0.037 (2)
C26	0.124 (4)	0.074 (3)	0.149 (4)	−0.026 (3)	0.099 (3)	−0.027 (3)
C27	0.093 (3)	0.057 (2)	0.068 (2)	0.017 (2)	0.039 (2)	0.0083 (18)
C28	0.152 (4)	0.080 (3)	0.099 (3)	0.025 (3)	0.076 (3)	0.009 (2)
C29	0.089 (3)	0.070 (3)	0.104 (3)	−0.001 (2)	0.034 (3)	−0.035 (2)
C30	0.067 (2)	0.063 (2)	0.101 (3)	−0.009 (2)	0.032 (2)	−0.022 (2)
C31	0.0419 (16)	0.0578 (18)	0.0433 (15)	0.0030 (14)	0.0124 (13)	−0.0024 (14)
C32	0.085 (3)	0.131 (4)	0.0451 (18)	0.018 (3)	0.0262 (18)	0.007 (2)
C33	0.076 (3)	0.136 (4)	0.059 (2)	0.022 (3)	0.030 (2)	−0.014 (3)
C34	0.077 (3)	0.130 (4)	0.093 (3)	0.008 (3)	0.033 (2)	−0.053 (3)
C35	0.079 (3)	0.074 (3)	0.099 (3)	0.019 (2)	0.031 (2)	−0.017 (2)

### Geometric parameters (Å, °)

**Table:**

Sn—O1	2.0716 (19)	C16—C17	1.362 (5)
Sn—C13	2.121 (3)	C16—H16	0.9300
Sn—C1	2.128 (3)	C17—C18	1.381 (4)
Sn—C7	2.135 (3)	C17—H17	0.9300
S1—C31	1.741 (3)	C18—H18	0.9300
S1—C32	1.817 (3)	C19—C20	1.490 (4)
S2—C31	1.768 (3)	C20—C31	1.361 (4)
S2—C35	1.813 (3)	C20—C21	1.479 (4)
C19—O1	1.300 (3)	C21—C22	1.468 (4)
C19—O2	1.224 (3)	C22—C23	1.329 (4)
C21—O3	1.219 (3)	C22—H22	0.9300
C1—C6	1.381 (4)	C23—C24	1.454 (4)
C1—C2	1.383 (4)	C23—H23	0.9300
C2—C3	1.378 (5)	C24—C25	1.370 (4)
C2—H2	0.9300	C24—C30	1.371 (4)
C3—C4	1.356 (5)	C25—C26	1.372 (5)
C3—H3	0.9300	C25—H25	0.9300
C4—C5	1.374 (6)	C26—C27	1.361 (5)
C4—H4	0.9300	C26—H26	0.9300
C5—C6	1.374 (5)	C27—C29	1.356 (5)
C5—H5	0.9300	C27—C28	1.504 (5)
C6—H6	0.9300	C28—H28A	0.9600
C7—C12	1.393 (4)	C28—H28B	0.9600
C7—C8	1.395 (4)	C28—H28C	0.9600
C8—C9	1.368 (4)	C29—C30	1.382 (5)
C8—H8	0.9300	C29—H29	0.9300
C9—C10	1.371 (5)	C30—H30	0.9300
C9—H9	0.9300	C32—C33	1.516 (5)
C10—C11	1.358 (5)	C32—H32A	0.9700
C10—H10	0.9300	C32—H32B	0.9700
C11—C12	1.385 (5)	C33—C34	1.472 (5)
C11—H11	0.9300	C33—H33A	0.9700
C12—H12	0.9300	C33—H33B	0.9700
C13—C14	1.389 (4)	C34—C35	1.519 (5)
C13—C18	1.390 (4)	C34—H34A	0.9700
C14—C15	1.380 (5)	C34—H34B	0.9700
C14—H14	0.9300	C35—H35A	0.9700
C15—C16	1.359 (5)	C35—H35B	0.9700
C15—H15	0.9300		
			
O1—Sn—C13	107.27 (10)	O1—C19—C20	117.0 (2)
O1—Sn—C1	110.16 (9)	C31—C20—C21	123.6 (3)
C13—Sn—C1	120.34 (11)	C31—C20—C19	120.0 (3)
O1—Sn—C7	93.93 (9)	C21—C20—C19	116.4 (2)
C13—Sn—C7	110.09 (11)	O3—C21—C22	121.6 (3)
C1—Sn—C7	111.75 (11)	O3—C21—C20	120.6 (3)
C31—S1—C32	105.85 (17)	C22—C21—C20	117.7 (3)
C31—S2—C35	104.38 (17)	C23—C22—C21	121.9 (3)
C19—O1—Sn	109.70 (17)	C23—C22—H22	119.0
C6—C1—C2	117.7 (3)	C21—C22—H22	119.0
C6—C1—Sn	119.2 (2)	C22—C23—C24	128.7 (3)
C2—C1—Sn	123.0 (2)	C22—C23—H23	115.6
C3—C2—C1	121.5 (3)	C24—C23—H23	115.6
C3—C2—H2	119.3	C25—C24—C30	115.8 (3)
C1—C2—H2	119.3	C25—C24—C23	123.7 (3)
C4—C3—C2	119.7 (4)	C30—C24—C23	120.5 (3)
C4—C3—H3	120.1	C24—C25—C26	121.5 (3)
C2—C3—H3	120.1	C24—C25—H25	119.3
C3—C4—C5	120.0 (4)	C26—C25—H25	119.3
C3—C4—H4	120.0	C27—C26—C25	122.9 (4)
C5—C4—H4	120.0	C27—C26—H26	118.6
C4—C5—C6	120.4 (4)	C25—C26—H26	118.6
C4—C5—H5	119.8	C29—C27—C26	116.0 (3)
C6—C5—H5	119.8	C29—C27—C28	122.0 (4)
C5—C6—C1	120.6 (3)	C26—C27—C28	122.1 (4)
C5—C6—H6	119.7	C27—C28—H28A	109.5
C1—C6—H6	119.7	C27—C28—H28B	109.5
C12—C7—C8	117.5 (3)	H28A—C28—H28B	109.5
C12—C7—Sn	121.6 (2)	C27—C28—H28C	109.5
C8—C7—Sn	120.8 (2)	H28A—C28—H28C	109.5
C9—C8—C7	121.3 (3)	H28B—C28—H28C	109.5
C9—C8—H8	119.3	C27—C29—C30	121.9 (4)
C7—C8—H8	119.3	C27—C29—H29	119.0
C8—C9—C10	120.2 (3)	C30—C29—H29	119.0
C8—C9—H9	119.9	C24—C30—C29	122.0 (4)
C10—C9—H9	119.9	C24—C30—H30	119.0
C11—C10—C9	119.9 (3)	C29—C30—H30	119.0
C11—C10—H10	120.0	C20—C31—S1	122.0 (2)
C9—C10—H10	120.0	C20—C31—S2	117.3 (2)
C10—C11—C12	120.8 (3)	S1—C31—S2	120.63 (16)
C10—C11—H11	119.6	C33—C32—S1	116.9 (3)
C12—C11—H11	119.6	C33—C32—H32A	108.1
C11—C12—C7	120.3 (3)	S1—C32—H32A	108.1
C11—C12—H12	119.9	C33—C32—H32B	108.1
C7—C12—H12	119.9	S1—C32—H32B	108.1
C14—C13—C18	118.0 (3)	H32A—C32—H32B	107.3
C14—C13—Sn	121.8 (2)	C34—C33—C32	114.9 (3)
C18—C13—Sn	120.1 (2)	C34—C33—H33A	108.5
C15—C14—C13	120.1 (3)	C32—C33—H33A	108.5
C15—C14—H14	120.0	C34—C33—H33B	108.5
C13—C14—H14	120.0	C32—C33—H33B	108.5
C16—C15—C14	120.8 (3)	H33A—C33—H33B	107.5
C16—C15—H15	119.6	C33—C34—C35	116.2 (3)
C14—C15—H15	119.6	C33—C34—H34A	108.2
C15—C16—C17	120.4 (4)	C35—C34—H34A	108.2
C15—C16—H16	119.8	C33—C34—H34B	108.2
C17—C16—H16	119.8	C35—C34—H34B	108.2
C16—C17—C18	119.7 (4)	H34A—C34—H34B	107.4
C16—C17—H17	120.2	C34—C35—S2	115.8 (3)
C18—C17—H17	120.2	C34—C35—H35A	108.3
C17—C18—C13	121.0 (3)	S2—C35—H35A	108.3
C17—C18—H18	119.5	C34—C35—H35B	108.3
C13—C18—H18	119.5	S2—C35—H35B	108.3
O2—C19—O1	121.5 (3)	H35A—C35—H35B	107.4
O2—C19—C20	121.5 (3)		
			
C13—Sn—O1—C19	−71.56 (19)	C16—C17—C18—C13	1.7 (5)
C1—Sn—O1—C19	61.1 (2)	C14—C13—C18—C17	−0.7 (5)
C7—Sn—O1—C19	175.99 (18)	Sn—C13—C18—C17	−178.1 (3)
O1—Sn—C1—C6	114.2 (2)	Sn—O1—C19—O2	5.6 (3)
C13—Sn—C1—C6	−120.2 (2)	Sn—O1—C19—C20	−174.50 (19)
C7—Sn—C1—C6	11.2 (3)	O2—C19—C20—C31	−109.5 (3)
O1—Sn—C1—C2	−67.2 (3)	O1—C19—C20—C31	70.6 (4)
C13—Sn—C1—C2	58.3 (3)	O2—C19—C20—C21	68.6 (4)
C7—Sn—C1—C2	−170.2 (2)	O1—C19—C20—C21	−111.2 (3)
C6—C1—C2—C3	−1.8 (5)	C31—C20—C21—O3	18.6 (5)
Sn—C1—C2—C3	179.6 (3)	C19—C20—C21—O3	−159.5 (3)
C1—C2—C3—C4	0.9 (6)	C31—C20—C21—C22	−160.3 (3)
C2—C3—C4—C5	−0.1 (6)	C19—C20—C21—C22	21.6 (4)
C3—C4—C5—C6	0.3 (7)	O3—C21—C22—C23	5.3 (5)
C4—C5—C6—C1	−1.3 (6)	C20—C21—C22—C23	−175.9 (3)
C2—C1—C6—C5	2.0 (5)	C21—C22—C23—C24	179.1 (3)
Sn—C1—C6—C5	−179.4 (3)	C22—C23—C24—C25	2.8 (5)
O1—Sn—C7—C12	120.0 (3)	C22—C23—C24—C30	−174.6 (3)
C13—Sn—C7—C12	10.0 (3)	C30—C24—C25—C26	0.3 (6)
C1—Sn—C7—C12	−126.5 (3)	C23—C24—C25—C26	−177.2 (4)
O1—Sn—C7—C8	−57.0 (3)	C24—C25—C26—C27	−0.5 (7)
C13—Sn—C7—C8	−167.0 (2)	C25—C26—C27—C29	0.6 (7)
C1—Sn—C7—C8	56.5 (3)	C25—C26—C27—C28	180.0 (4)
C12—C7—C8—C9	0.4 (5)	C26—C27—C29—C30	−0.7 (6)
Sn—C7—C8—C9	177.5 (3)	C28—C27—C29—C30	180.0 (4)
C7—C8—C9—C10	0.2 (6)	C25—C24—C30—C29	−0.4 (6)
C8—C9—C10—C11	−0.8 (7)	C23—C24—C30—C29	177.2 (3)
C9—C10—C11—C12	0.8 (6)	C27—C29—C30—C24	0.7 (6)
C10—C11—C12—C7	−0.2 (6)	C21—C20—C31—S1	−2.8 (4)
C8—C7—C12—C11	−0.4 (5)	C19—C20—C31—S1	175.2 (2)
Sn—C7—C12—C11	−177.5 (3)	C21—C20—C31—S2	174.4 (2)
O1—Sn—C13—C14	128.4 (2)	C19—C20—C31—S2	−7.6 (4)
C1—Sn—C13—C14	1.6 (3)	C32—S1—C31—C20	156.1 (2)
C7—Sn—C13—C14	−130.6 (2)	C32—S1—C31—S2	−21.1 (2)
O1—Sn—C13—C18	−54.3 (3)	C35—S2—C31—C20	138.4 (2)
C1—Sn—C13—C18	178.8 (2)	C35—S2—C31—S1	−44.3 (2)
C7—Sn—C13—C18	46.6 (3)	C31—S1—C32—C33	86.2 (3)
C18—C13—C14—C15	0.1 (5)	S1—C32—C33—C34	−83.9 (4)
Sn—C13—C14—C15	177.3 (2)	C32—C33—C34—C35	58.3 (5)
C13—C14—C15—C16	−0.4 (5)	C33—C34—C35—S2	−76.7 (4)
C14—C15—C16—C17	1.4 (6)	C31—S2—C35—C34	91.1 (3)
C15—C16—C17—C18	−2.0 (6)		
